# Introduction—American Balneology

**Published:** 1916-11

**Authors:** Felix Von Oefele

**Affiliations:** New York


### Introduction—American Balneology

DR. FELIX VON OEFELE, New York.

Chemically pure water can be produced only by the reaction of pure hydrogen and pure oxygen in the chemical laboratory, as the well known Professor Oscar Loew (a born American scientist) did in large quantities for his sugar syntheses at Munich. We will find his name with the investigators of California mineral springs. Water is retained pure with difficulty, is a colorless, odorless and tasteless fluid and has a high solvent power for many chemical compounds. An almost pure water is the so-called distilled water, purified by evaporation and recondensation. It is similar to rain water, but still purer. The rain water is distilled by nature and absorbs some vapors and suspensions of the air, from which the artificially and properly distilled water is protected.

Water, as it falls from the clouds in the form of rain, absorbs particularly various gases, impurities of the air, so that when obtained in its purest natural state, it contains Oxygen, Nitrogen, Carbonic acid and sometimes traces of carburated Hydrogen, Sulphur dioxyde, Nitric acid and Ammonia. Having fallen upon the earth the water penetrates the soil and rocks of the surface of the earth and it may lose some part of its contents, but takes up various other salts and organic substances from the soil. It reappears earlier or later on the surface of the earth. A place where such water appears continuously is called a spring and, where it does so by human artificial means, a well.

As to their occurrence, springs may be divided into four classes: (1) Natural drift springs, (2) natural stratum springs, (3) natural fault springs, and (4) artificial wells including largely the artesian wells.

The common well is an artificial drift spring. The artesian well is an artificial stratum spring. Artesian wells are created artificially by tapping a continuous water bearing stratum in the bowels of the earth. The water from it flows by natural hydraulic pressure. If surface water is collected in a hole with water proof walls, we call it a cistern. The cistern keeps the water cooler and purer from germs than a surface reservoir. If an artificial hole within the drift or below the drift collects the water dripping from the surroundings, we call this a well.

Since drift is the uppermost brittle layer of the earth's crust, the water of drift springs did not penetrate the solidified rocks. The waters of a stratum spring pass through a number of layers of deposited solidified rocks, before they again reach the surface through the medium of the lowest stratum, which the water had reached. Fault springs are due to a disturbance of the regularity of the earth's strata by reason of a fracture. In such instances, what was originally one continuous stratum is broken up and presents disturbed strata on both sides of the fault. In fault springs, the entire water or a large percentage of it often rises in a column or ribbon above the level of the earth and consists of a large amount of magmatic water.

Many individual springs arise everywhere upon the surface of the earth. Only in the deserts and in Egypt are springs very rare.

All spring waters, having been in contact with different strata of the earth, hold in solution variable quantities of anorganic compounds, the nature and quantity of which depend upon the geology and petrography of the region. Therefore every spring water contains some carbonic acid, sodium, calcium, chlorine, sulfuric acid and silicon. The same constituents are the anorganic matter of the human serum and of all the physiological liquids of animals and plants. Average springs contain far smaller quantities of the rarer constituents for instance Potassium, Magnesium, Bromine, Iodine, Iron, Aluminium and other ones. Both these groups of constituents have quantitatively different valencies as physiological agents. We must differentiate them into the invalid and valid group.

Some water may be used for technical purposes, but not as desirable drinking water for the human being. Other water may be good enough for drinking purposes, but not for certain technical purposes. Some waters are good in both regards, some are not good in either regard. And, also, these four groups are not separable by strict boundaries. Different species of animals and different races of human beings depend to a different degree upon pure drinking water. The absence of water forms the desert. Where water appears within the desert the life of plants and animals starts. The standard of a useful drinking water is unrestricted there. But where large cities are situated on the margin of mountains with manifold development of different geological strata, aqueducts have been constructed for thousands of years. The city Assur of old Mesopotamia is an example. The best resources of water are selected. Later the emperors of old Rome supplied the city with streams of the best water. Not theory, but practical experience showed in old times the best waters of drinking purposes, domestic use and technical needs for these large cities.

Every spring water may be used under certain conditions for therapeutic purposes. It depends upon circumstances, if this or that water is preferred. Every water may be used in exceptional cases as a medicinal water. This proves the impossibility to separate absolutely medicinal waters and common waters. The highly mineralized waters are rejectionable for common use, but show clear pharmakodynamic properties. The low mineralized waters are desirable drinking waters. Some are more, some less desirable. This desirability may also change according to circumstances. The wealthy man may use a more desirable bottled water instead of the common water of his residence by his own selection. That is a table water.

For more than two decades the Chalybeate Water of Sullivan County, New York, was very little used for therapeutic purposes with medical advice. I myself made the first chemical analysis and interested the medical profession. Some physicians have sent an increasing number of suitable patients there. The proprietor did not acknowledge the advice of experienced practitioners. The local lay people tried to invent their own methods of application in order to save money and increase the profits. The medical profession will not patronize such a spring while under such control. Mistakes lasting years or even decades or even centuries may be overlooked, if the spring eventually comes into the right hands. During the mistakes is it not a useful medicinal spring; after the rectification it will again become a useful medicinal spring and the patronage of the medical profession will return at the same time.

The physician may be enforced by circumstances to prohibit the use of the residence water and to recommend certain table waters. There the table water begins insensibly to become a medicinal water. No strict limits exist between common useful water and table water and medicinal water.

The occasional recommendation of the physician may include the best waters of neighboring townships. A small township may select from a number of springs of the vicinity the least objectionable water and neighboring townships with worse water supply may estimate such water as table water or more as relative medicinal mineral spring.

The definition of good drinking waters, domestic waters and technical waters is developed by experience and can only be given by the collection of properties and comparison of old recognized waters.

### DEFINITION OF MEDICINAL MINERAL SPRINGS.

There are many thousand natural springs in the United States, of which however, only 782 are reported to have in 1911 actual sales of water. The number in other years is not much different. These springs include table waters and medicinal waters; more than two thirds of the money value of these sales are from table waters.

American country mineral springs are little known and are less used by the medical profession. But innumerable American springs may be used therapeutically for the benefit of many sick people.

The question has often arisen, as to what a medicinal mineral spring is, and as such what its action upon the ailing or sickly is. A mineral spring is a body of natural spring water containing a certain amount of dissolved minerals in such quantities as to exert a physiological action upon the human body, and may be capable of being useful by its special chemical or physical constitution for therapeutic purposes in treatment of sick people besides augmenting the supply of water to the blood and tissues. The therapeutic uses of such waters depend upon the nature or properties of the mineral matter present or absent. This constitutes the difference between medicated (mineral) waters and ordinary (potable) water. The European Appolinaris water is no medicinal mineral spring in two principal regards. It is not a natural spring water and is only used as luxurious table water without therapeutic intention.

The action upon the sick people is not as readily answered. First because there are so many different varieties of mineral springs; secondly it entails a knowledge of the particular spring in question and an authentic, scientific analysis of the springs contents.

This is the result of a historical development. A high percentage of nomadic and highly cultivated countries since time immemorial have semi-annually or oftener changed their residences. The prominent people lived in cities in the winter season and in summer season in the country. The increased need of cities enforced larger water supplies. The different influence between well or river water of the city and of country spring water was early noticed. Sick people used the summer resorts of friends with more fitting spring water, it was a primitive mineral spring cure. A special issue of the Hippocratic collection concerns the influences of different climates, waters and locations. The continuous comparisons with Egypt and some time with Macedonic conditions prove the book as an original very old Egyptian book translated and adapted to the country and time of Alexander the Great. The hunting tribes of the Indians changed the summer and winter resorts through necessity resulting from primitive food supply without the development of cities.

The useful water of the single case may have been purer or lower mineralized or may have contained a larger amount of pharmakodynamic constituents or properties. As stated above, the dissolved mineral constituents must be divided into valid and invalid ones. The action of the valid constituents depends less upon their present amount and is pharmakodynamic. The action of the invalid constituents depends upon a present excess and is physicodynamic.

The undoubtedly medicinal water starts, when the trace of this or that valid constituent of a low mineralized water passes the boundary of pharmakodynamic action. A low mineralized spring may contain particular valid constituents in a relatively high percentage. The valid constituents furnish subdivisions of low mineralized waters in chemical regard. Silicon and Magnesia stand on the boundary of invalid and valid constituents. Invalid constituents may only create classification of mineral springs of supermineralized character.

Medicinal mineral springs may differ from common springs in one or more of the following properties: 1) in temperature, 2) in an uncommonly prevalent amount of one or more of the rarer constituents which we call the valid ones, 3) in abnormal low or 4) high amount of one or more of the common constituents which we called the invalid ones and 5) in a differing pressure of gases. These classes of springs are named according to their content of constituents.

In the majority of waters, an analysis has not been done upon the American springs. There are many widely advertised mineral springs whose owners claim them to contain certain valuable ingredients of great medicinal value of the valid group and capable of alleviating and curing all manner of diseases. This in reality, they are incapable of doing, because the efficiency of every spring is limited to few symptoms important for a particular group of diseases. It is not because the owners are dishonest and wish to hoodwink the public, but because their knowledge of the springs contents is what they have learned by empiricism and by expansion of this empiricism by flattering friends without criticism, then again by some arbitrary analysis claiming indefinitely faint trace of some valid constituent and not by a careful and scientific one.

---

**Epidemic Gonorrhoea.** Bendig of Stuttgart, Muench. Med. Woch., describe epidemics involving two institutions for girls. One girl aged 8, was the origin of the disease in 15 of 40 inmates, due to the custom of having two girls bathe together and use the same towel. There was ophthalmia, endocarditis or perimetritis but the course of the cases was prolonged, from 10 weeks to 279 days in one case. Treatment consisted in santal oil internally, irrigations of potassium permanganate and protargol and swabbing with ichthyol..

